# Gamification of dermatoscopy education using a smartphone mobile platform: A pilot study

**DOI:** 10.1016/j.jdin.2024.03.008

**Published:** 2024-04-20

**Authors:** Shashendra Aponso, Yue Ting Nichole Tan, Siddharth Jain, Choon Chiat Oh

**Affiliations:** a Department of Dermatology, Singapore General Hospital, Singapore, Singapore; b Duke-National University of Singapore Medical School, Singapore, Singapore; c Playware Studios Pte Ltd, Singapore, Singapore

**Keywords:** dermatoscopy, dermoscopy, education, gamification, mobile, smartphone

## Abstract

**Background:**

Dermatoscopy is a noninvasive method of examining skin lesions under high magnification, gradually replacing the need for invasive biopsies. Training is required to gain clinical competency. Gamification employs game-like elements to enhance education engagement and is an engaging means of delivering medical education. We sought to use gamification and a mobile-based platform to deliver dermatoscopy education to physicians.

**Methods:**

We developed SKIN@GoPRIME, an interactive smartphone platform. Thirty physician participants were randomly assigned to watch an online dermatoscopy lecture or to use SKIN@GoPRIME. Twenty-eight participants completed prelearning and postlearning quizzes and provided feedback on SKIN@GoPRIME.

**Results:**

Users of SKIN@GoPRIME demonstrated a significant 1.71-point mean score improvement (*P* = .0018). The group that watched the online dermatoscopy lecture had a higher 2.36-point mean score improvement (*P* = .00021). Both family medicine and internal medicine physicians demonstrated a significant mean score increase of 1.29 (*P* = .049) and 2.14 (*P* = .023), respectively, after using SKIN@GoPRIME. Based on feedback, 83% believed that SKIN@GoPRIME can be used to acquire the applied competencies required for their job scope.

**Discussion and Conclusion:**

SKIN@GoPRIME, a novel learning tool via gamification effectively delivers dermatoscopy education, although it is not shown to be more effective than lectures. Larger studies are required to further validate the effectiveness of gamified learning techniques in dermatoscopy education. Future studies should involve the optimization of SKIN@GoPRIME to more effectively deliver dermatoscopy education.

**Capsule Summary**

• With gamification, we developed a novel smartphone platform, SKIN@GoPRIME, aligned with changing models of education in an increasing digitalized world.
• SKIN@GoPRIME makes dermatoscopy education accessible and convenient, although it is not shown to be better than learning dermatoscopy via lecture.

## Introduction

Gamification is a modern and attractive learning modality involving games, applications, and virtual scenarios. It empowers users to take charge of their own learning and provides continuous motivation and feedback to reinforce the habit of learning.^1^ Increasingly, gamification has been utilized in medical dermatology education and can complement or serve as an alternative approach to traditional methods of learning.^2^ However, an unmet need in the teaching of dermatology is an effective and engaging means of delivering dermatoscopy education.

In Singapore, skin cancer remains one of the top 10 most frequently diagnosed cancers and has displayed an upward trend over the past 5 decades.^3^ Patients often first present to their general care practitioner who must be cognizant of a growing proportion of patients who may present with skin cancer. Dermatoscopy is a portable bedside tool that has demonstrated to be an effective tool in the triage of suspicious skin lesions in the primary care setting.^4^ However, training is required to gain competency in the use of dermatoscopy and physicians often cite a lack of training as a key barrier to its use.^5^^,^^6^ There is a need to empower physicians with the skills required to effectively use dermatoscopy in order to facilitate the detection of skin cancer in the community. To date, most dermatoscopy training programs make use of traditional methods of education.^7^ Hence, there is potential to explore the role and effectiveness of gamification in delivering dermatoscopy education.

As such, we sought to develop an interactive smartphone mobile platform that taps on the elements of gamification to deliver dermatoscopy education. We also aimed to investigate if the platform can serve as an effective educational tool as compared with traditional methods of learning.

## Methods

SKIN@GoPRIME is a smartphone mobile platform developed by Playware Studios, a Singapore-based educational technology company that specializes in developing games and technology for teaching and learning. Incorporating the principles of gamification, it features bite-sized learning modules containing games and quizzes, allowing users to receive concise and relevant content from the convenience of their smartphones. The pilot version of the platform was made available on both web browser as well as Android/iOS mobile devices as a smartphone application.

Once learners logged in to their accounts, they arrived at a homepage menu that enabled them to access the various features of the platform; self-assessments, progress board, notifications, a journal, and the learning community features (namely a forum and an events calendar). The self-assessment section provided learners with 2 options, game-based learning and quizzes, and learners were given the flexibility to start with either section. Game-based learning consisted of 4 animated clinical simulation scenarios featuring a clinic doctor attending to a patient with skin lesion. As each scenario progressed, there were built-in pauses during which the learner was required to make a clinical decision or answer a dermatoscopy question. For example, when learner had chosen a lesion to examine, they were provided with a clinical photograph and a zoomed in dermatoscopic photograph on which they had to identify relevant dermatoscopic features and finally classify the lesion as benign or malignant. Learners scored points for each correct answer, motivating them to perform better. Game scores were revealed at the end of each scenario.

On the other hand, the quiz section consisted of a variety of dermatoscopy knowledge-based and image-based questions. Format of the questions included multiple choice questions, matching the correct word from a list and correctly identifying/marking out dermatoscopic features on an image.

The platform was designed to foster collaborative and active learning by creating avenues for peer feedback and review. With this aim in mind, one of the featured sections was a discussion forum, where learners were able to post questions and topics that they found challenging. Learners received notifications when other users or the platform (either fellow learners or dermatoscopy context experts) responded to their questions.

Furthermore, the platform features virtual bedside rounds to facilitate interactive and engaging teaching and learning experiences with mentors. These are real, case-based discussions with mentors, wherein select dermatoscopy images and their clinical context are discussed by the dermatoscopy content expert and learners can ask questions. This also allows for real-time remote learning that mimics an in-room experience and allows mentors to closely monitor users’ progress. Lastly, the platform features a virtual Balint group to allow users to discuss challenging dermatoscopy cases, further promoting collaborative learning and enabling users to develop a better understanding and awareness of the doctor-patient relationship.^8^^,^^9^

Learners were given 1 month to engage with the platform and were provided with required technical assistance via email/telephone during office hours. The technical team was able to extract learner login times as well as the time of completion of the various features of the platform. Hence, we were able to determine whether learners used the platform as they should.

Participants were physicians recruited from 2 specialties, namely from family medicine (residents as well as general practitioners/family physicians) and internal medicine (residents). Of the 30 participants, 15 were randomly assigned to watch a lecture video on dermatoscopy, and 15 were randomly assigned to use SKIN@GoPRIME. The dermatoscopy lecture video was a one and a half hour video and focused on clinical photographs and dermatoscopic photographs of various skin lesions to illustrate dermatoscopic features.

**Fig 1:**
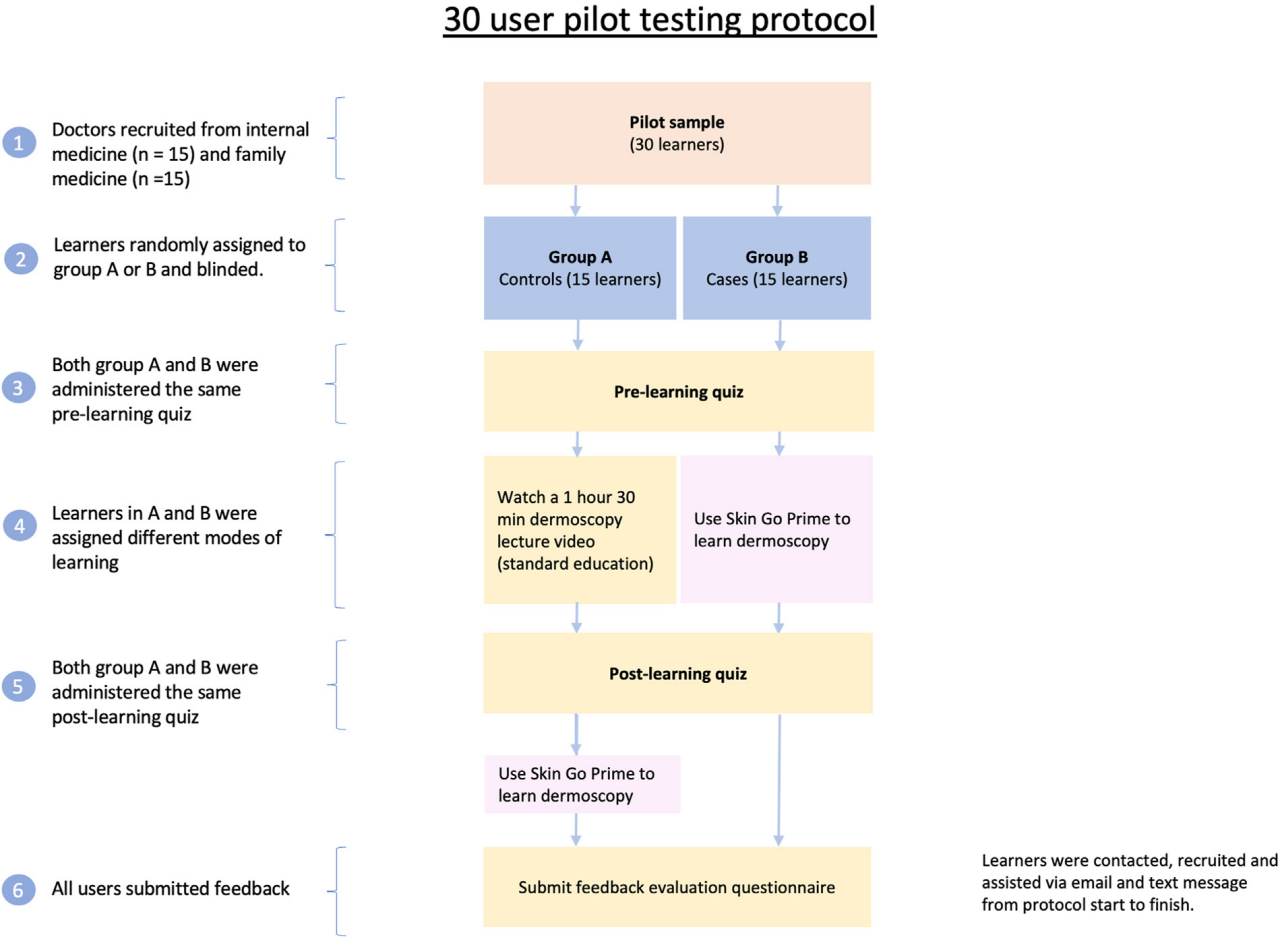
User testing protocol.

To assess the participants’ knowledge, a dermatoscopy quiz was administered before and after watching the lecture and using SKIN@GoPRIME. The quiz consisted of 5 image-based and 5 knowledge-based questions where users had to fill in the blanks by choosing the correct word from a list of 30 possible answers. The same quiz was used before learning and after learning. The postlearning quiz was administered 1 month after the prelearning quiz, giving the learners adequate time to access the learning material. None of the images or questions in the quiz were duplicated in either of the educational material. Each correct answer was worth 1 point, hence the highest possible quiz score was 10. At the end of the study, all participants used SKIN@GoPRIME and feedback was collected in the form of an evaluation questionnaire (Fig 1). Statistical significance was calculated at *P* < .05 using paired and 2-sample *t* tests.

## Results

Among the 15 participants who were assigned to watch the lecture video, 14 completed both prelearning and postlearning quizzes. Likewise, 14 participants completed both quizzes among the 15 who were assigned to use SKIN@GoPRIME. All participants submitted their feedback via an evaluation questionnaire at the end of the study. The results of this study are summarized in Table I.

**Table I:** Participants and mean score improvement

Participants' speciality training	No. of participants	Mean prelearning quiz score	Mean postlearning quiz score	Mean score improvement	*P* value
Total participants					
Lecture video	14	5.64	8	2.36	.00021
SKIN@GoPRIME	14	4.71	6.43	1.71	.0018
Family medicine					
Total	14	5.29	7.43	2.14	
Lecture video	7	5.43	8.43	3	.0048
SKIN@GoPRIME	7	5.14	6.43	1.29	.049
Internal medicine					
Total	14	5.07	7	1.93	
Lecture video	7	5.86	7.57	1.71	.023
SKIN@GoPRIME	7	4.29	6.43	2.14	.023

Score improvement for each learner was calculated by subtracting prelearning test score from the postlearning test score. Mean score improvement was calculated for the 2 groups. The highest achievable test score was 10 out of 10. Overall, participants who used SKIN@GoPRIME demonstrated a 1.71-point mean score improvement (*P* = .0018). Participants who watched the video lecture demonstrated a higher 2.36-point mean score improvement (*P* = .00021). On further subgroup analysis, both family medicine and internal medicine physicians also demonstrated a significant mean score increase of 1.29 (*P* = .049) and 2.14 (*P* = .023) respectively, after using SKIN@GoPRIME. This indicates that SKIN@GoPRIME was successful in imparting dermatoscopy knowledge, although it is not shown to be better than learning dermatoscopy via lecture.

Based on user feedback, 83% believed that SKIN@GoPRIME could be used as a form of training to acquire the applied competencies required in their job scope. Among the top feedback comments included how the platform was interactive and engaging, accessible and convenient, and with realistic clinical scenarios and versatile modes of assessment (Table II).

**Table II:** Top user feedback

Positive feedback	Negative feedback
Interactive and engaging	Can be better tailored to target different learning groups (eg, doctors with no prior dermatology training may need more explanations)
Accessible and convenient	
Realistic clinical scenarios	
Versatile modes of assessment	

## Discussion and conclusion

Traditional dermatoscopy training involves didactic learning as well as hands-on training. In dermatology residency programs in the United States, didactic lectures were reported to be the most common dermatoscopy teaching modality, and few received dedicated training on the use of the dermatoscopy in evaluating skin lesions.^10^ In Singapore, dermatoscopy is a skill that is still largely limited to dermatologists. Although a dermatoscope is readily available, there is a lack of trainers in Singapore. Given the growing incidence of skin cancer in the population and keeping in line with the government’s initiative to shift care to primary care physicians, there is a greater need to equip primary care physicians, such as family medicine doctors, with the skills required to effectively triage skin lesions with a dermatoscope. As such, SKIN@GoPRIME takes a novel approach away from traditional methods of learning. Instead, it taps on the features of gamification and provides learners with an easy access to dermatoscopy education in a highly convenient manner, features simulations in a clinical setting, and facilitates interaction with the faculty via virtual bedside rounds and Balint groups. The platform is also designed to target medical professionals by delivering information succinctly and enticing learners with various learning options such as games and quizzes.

The results in our pilot study are in line with the current literature that gamified learning in medical education improves test scores, engagement, and satisfaction among learners.^1^ Although there have been a few applications and websites featuring gamified learning in dermatoscopy, none of them featured scenario games, which SKIN@GoPRIME provides.^1^

There are currently 3 popular mobile applications that deliver dermatoscopy education. The first is Dermloop Learn, an artificial intelligence driven training application (https://melatech.io/dermloop/). The key feature of Dermloop Learn is that as users interact with the application, their learning patterns are analyzed and compared against those of past users in order to predict and provide each user with an optimized training program. Additionally, it features dermatoscopy quizzes, a score board to allow users to compare scores with other users, and multiple levels of difficulty. The second dermatoscopy education application is YouDermoscopy, which features image-based quizzes of various skin lesions (https://www.youdermoscopytraining.org/). Again, there are different training levels (the developers claim that it is the first application divided into different training levels). Thirdly, the Dermoscopy Two Step Algorithm application features a series of cases with sequential questions to allow the learner to arrive at a diagnosis (https://apps.apple.com/iq/app/dermoscopy-two-step-algorithm/id731753300). There is no scoreboard hence users are unable to track the percentage of correct answers. SKIN@GoPRIME distinguishes itself from the other 3 applications as it is the only one that uses serious games/simulation scenarios to deliver education. The advantages that the other applications have over SKIN@GoPRIME are artificial intelligence–based personalization of learning and time constrains given to answer quizzes in Dermloop Learn and YouDermoscopy respectively. However, we believe that users are more likely to find SKIN@GoPRIME engaging for 2 main reasons. Users will feel more invested in the application when they play the role of the clinician in the serious games featured in this application. Secondly, SKIN@GoPRIME provides a learning community with virtual Balint groups in addition to discussion forums—learners will feel a sense of belonging to a learning community, increasing their desire to continue using the application.

The mean score improvement of participants who watched the lecture video was higher than those who used SKIN@GoPRIME. The reason might be due to a lack of access to learning materials in SKIN@GoPRIME. For the traditional learning group, all participants had to watch the lecture video on dermatoscopy, wherein the lecturer systematically walked the users through algorithms and the various basic dermatoscopic features. In contrast, SKIN@GoPRIME does not have any embedded lecture videos or background information regarding the various topics encountered in the platform. Compared with the traditional lecture video, SKIN@GoPRIME allowed users more flexibility in tailoring their learning to suit their time and interests; however, this may have come at the cost of a systematic/layered approach to knowledge consolidation. In addition, the platform did not have all the answers to the questions provided in the games. To improve the effectiveness of the platform, we plan to redesign SKIN@GoPRIME to include more features to further enhance learning. These features include embedding hyperlinks to allow users to access relevant learning materials as they progress through the games and quizzes, including flashcards, enabling daily notifications with bite-sized information, including varying levels of quiz complexities, and offering more simulations to reflect daily clinical practice.

SKIN@GoPRIME was developed as a tool to supplement, and not entirely replace traditional modes of dermatology education delivery such as didactic lectures. A didactic dermatoscopy lecture forces learners to sit down for a fixed time (eg, 2-3 hours) during which knowledge is gained in a structured manner. For instance, the lecture may sequentially focus on dermatoscopic features such as pigmented lesions, acral lesions, common vascular patterns, and so forth, systematically guiding the learner through the educational content. In contrast, SKIN@GoPRIME was designed to allow learners to access and consume bite-sized educational content on the go. Although the platform does not force the learner to dedicate a long period of time at once or provide a sequence in which to cover the material, it takes into account perhaps the most valuable commodity in busy doctor’s life—time. SKIN@GoPRIME’s strength lies in the fact that a resident doctor may use the platform to learn the features of a basal cell carcinoma during their train ride to work, or quiz themselves during a 15-minute break. Based on our user feedback, the platform also generates and maintains interest in learning. Therefore, through generating learner engagement, maintaining learner interest and delivering educational content in a more accessible format, SKIN@GoPRIME can add value even when used alone.

We acknowledge that there are several study limitations. Firstly, the small sample size of 30 learners (15 in each subgroup) may not fully capture the breadth of proficiencies or dermatologic exposure within the group of learners. As this was only a pilot study, we hope to recruit a larger sample of learners for subsequent studies of our learning platform. Secondly, there was no means of controlling for the amount of dermatology knowledge that the learners had. For instance, some doctors may have rotated through dermatology before whereas others may not have had any clinical dermatology education after medical school. Furthermore, we did not include surgical specialties or other medical specialties other than family medicine and internal medicine in this study. Finally, since this was a pilot version of the platform, only a limited number of questions and games were featured. The content covered by these limited questions and games may not have covered the breadth of information that was delivered in the prerecorded dermatology lecture that the learners in the comparison group watched.

We observed that when comparing test scores in the group that used SKIN@GoPRIME, internal medicine doctors performed better than family medicine. Internal medicine doctors demonstrated better learning outcomes after using SKIN@GoPRIME compared with the lecture video (mean score improvement of 2.14 compared with 1.29, respectively). Internal medicine doctors have a busy time schedule with overnight on-call duties and hence these results may reflect the fact that time-starved doctors prefer the use of mobile applications/learning platforms to learn on the go/between their night calls. This is in in contrast to family medicine doctors, whose training often involves lectures delivered over weekends. It highlights that the platform in its current form may be better suited for certain learners over others—an area that needs to be improved upon.

Ultimately, SKIN@GoPRIME is a learning tool and through its creation, we sought to design a novel method of delivering dermatoscopy education effectively to physicians. It is part of the paradigm shift in medical education which now emphasizes on gamified learning in an increasingly digitalized world. Delivery of education to physicians has to also take into account the busy schedule of having to learn while working in hospitals or in clinics, and therefore requires a more varied approach compared with traditional methods of learning. As such, and with the results from this pilot study, SKIN@GoPRIME is an attractive educational tool for the efficient and convenient learning of dermatoscopy.

Moving forward, larger studies are required to further validate the effectiveness of gamified learning techniques in dermatoscopy education. Future studies should also be involved in the optimization of SKIN@GoPRIME to deliver dermatoscopy education more effectively.

## Conflicts of interest

None disclosed.
